# Case Report on Spontaneous Coronary Artery Dissection: A Rare Culprit of Chest Pain

**DOI:** 10.7759/cureus.47645

**Published:** 2023-10-25

**Authors:** Aiman D Khudair, Thuraiya H Al-Rawahia, Rachel A Marshall, Ahmed D Khudair, Chetan Narayana, Leena Sulaibeekh

**Affiliations:** 1 Department of Cardiology, Royal College of Surgeons in Ireland - Bahrain, Muharraq, BHR; 2 Department of Cardiology, Mohammed Bin Khalifa Bin Salman Al Khalifa Specialist Cardiac Centre, Riffa, BHR

**Keywords:** coronary angiography, non st-elevation myocardial infarction, chest pain, acute coronary syndrome, spontaneous coronary artery dissection

## Abstract

Spontaneous coronary artery dissection (SCAD) is a non-atherosclerotic separation of the coronary artery wall with subsequent intramural hematoma (IMH) formation in the false lumen. It can be associated with or without an intimal tear. It is clinically divided into three types based on its angiographic appearance. Most SCAD cases are seen in young or middle-aged women, especially in a peripartum state. Additionally, SCAD patients usually have fewer cardiovascular risk factors and more commonly have predisposing conditions like fibromuscular dysplasia (FMD). Patients present with features of chest pain that radiates to the left arm or neck, shortness of breath (SOB), as well as nausea and vomiting. Coronary angiography is the most widely used first-line modality to diagnose this condition. Management is usually conservative; however, invasive procedures can be utilized for high-risk patients. We present a case of a 54-year-old woman with SCAD diagnosed using coronary angiography and treated conservatively with dual-antiplatelet therapy, culminating with resolution.

## Introduction

Spontaneous coronary artery dissection (SCAD) is a rare cause of acute coronary syndrome (ACS) [1]. It arises from a spontaneous non-atherosclerotic separation of the coronary artery wall, producing an intramural hematoma (IMH) in the false lumen, which can also be associated with an intimal tear. Patients clinically present with shortness of breath (SOB) and acute severe chest pain that radiates to the left arm or neck, diaphoresis, and palpitations [2,3]. There is a higher clinical index of suspicion for SCAD in young or middle-aged women presenting with myocardial infarction (MI), especially in the peripartum period. In addition, it can manifest in patients with the absence of classic cardiovascular risk factors. SCAD less frequently presents in men but commonly occurs following recent intensive emotional or physical stress. Predisposing conditions like fibromuscular dysplasia (FMD) have also been reported to contribute to the increased risk of SCAD [4]. SCAD can be classified into three types based on its angiographic appearance [2]. Our case is a middle-aged woman who presented with a non-ST elevation myocardial infarction (NSTEMI) secondary to type 2A SCAD and was diagnosed using CT coronary angiography.

## Case presentation

**Figure 1:**
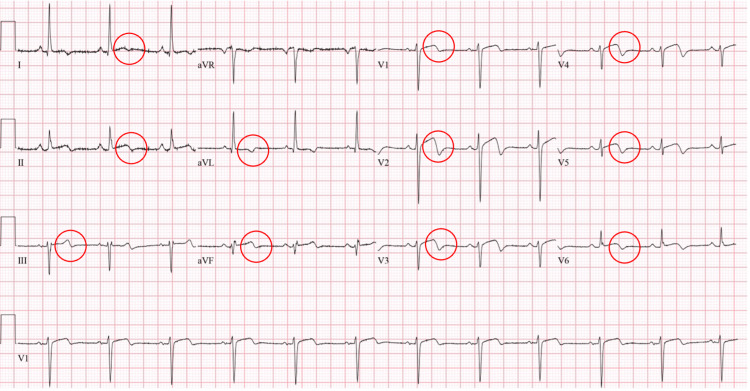
ECG upon admission for a female patient with type 2A SCAD of the LAD artery, showing normal sinus rhythm and biphasic T waves in leads V1 to V6, L1, L2, L3, aVF, and aVL (circles), consistent with NSTEMI. ECG: Electrocardiogram; SCAD: Spontaneous coronary artery dissection; LAD: Left anterior descending; NSTEMI: Non-ST-elevation myocardial infarction.

A 54-year-old female presented to the ED of a secondary care hospital after experiencing a single episode of burning, non-exertional chest pain. She reported that the intense pain lasted for approximately 20 to 30 minutes. Her high-sensitivity troponin levels were elevated at 1,600 ng/L, and an ECG displayed subtle biphasic T-waves in leads V1 to V6, L1, L2, L3, aVF, and aVL, findings consistent with NSTEMI. She was subsequently administered aspirin. Further investigations revealed anemia with a hemoglobin level of 70 g/L (normal range: 120-160), which was addressed with a transfusion of three units of packed RBCs. An esophagogastroduodenoscopy revealed mild gastritis, while a colonoscopy showed no active source of bleeding. Three days later, she was transferred to a specialist cardiac center for coronary angiography. Her past medical history is significant for mild gastritis, iron deficiency anemia, and menorrhagia for two years. However, upon presentation, she was not actively bleeding. There was no history of hypertension, diabetes mellitus, dyslipidemia, smoking, or family history of premature coronary artery disease. The patient was not on any medications and had no known allergies. On admission at our specialist cardiac center, she was clinically and vitally stable with a regular pulse of 75 beats per minute, a blood pressure of 121/59 mmHg, afebrile, and a recorded BMI of 34.62 kg/m^2^. Chest and lung examinations were unremarkable. Cardiac auscultation revealed no appreciable heart murmurs. Laboratory tests were normal except for low hemoglobin with a hypochromic microcytic blood picture and mildly elevated troponin-I (Table 1). ECG was performed and revealed normal sinus rhythm and biphasic T wave in leads V1 to V6, L1, L2, L3, aVF, and aVL (Figure 1).

**Table 1:** Laboratory results for a patient with type 2A spontaneous coronary artery dissection in the left anterior descending artery upon admission.

Laboratory investigations	Reference ranges	Level
Creatinine (µmol/L)	44-80	41
Hemoglobin (g/L)	120-160	94.1
Hematocrit (L/L)	0.35-0.45	0.304
RBCs (L/L)	3.8-4.8	5.18
Mean corpuscular volume (fl)	78-100	58.7
Mean corpuscular hemoglobin (pg)	27-32	18.2
Red cell distribution width (%)	11.5-16	33.9
Mean platelet volume (fl)	6-9.5	10.72
Troponin-I (ng/mL)	0.01-0.04	0.078
Creatine kinase-myocardial band (µg/L)	0-3.6	1
Creatine kinase (IU/L)	20-180	22

**Figure 2:**
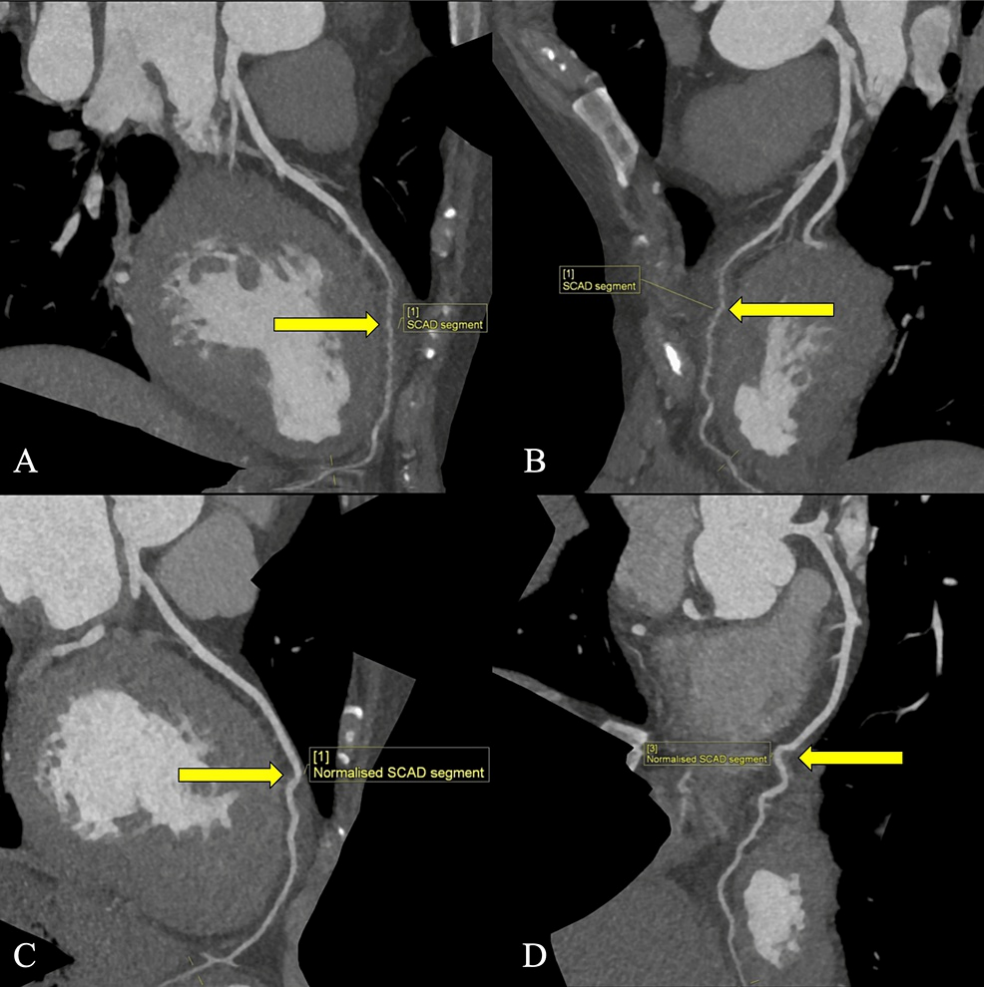
A, B: CT coronary angiogram showing type 2A SCAD upon admission, characterized by abrupt, long-segment narrowing in the middle segment of the LAD artery extending to the distal segment (arrows); C, D: Follow-up imaging after eight months, displaying good caliber in the middle segment of the LAD artery and suggesting resolution of SCAD (arrows). SCAD: Spontaneous coronary artery dissection; LAD: Left anterior descending.

The following day, a CT coronary angiography was performed. It revealed an abrupt and progressively significant narrowing in the middle segment of the left anterior descending (LAD) artery, extending over a significant length (>20mm) to its distal segment. However, the segment near the left ventricular apex was spared. The right coronary artery and left circumflex coronary arteries were free from atherosclerotic disease or luminal narrowing. These findings are suggestive of type 2A SCAD involving the LAD artery (Figure 2A, 2B).

**Figure 3:**
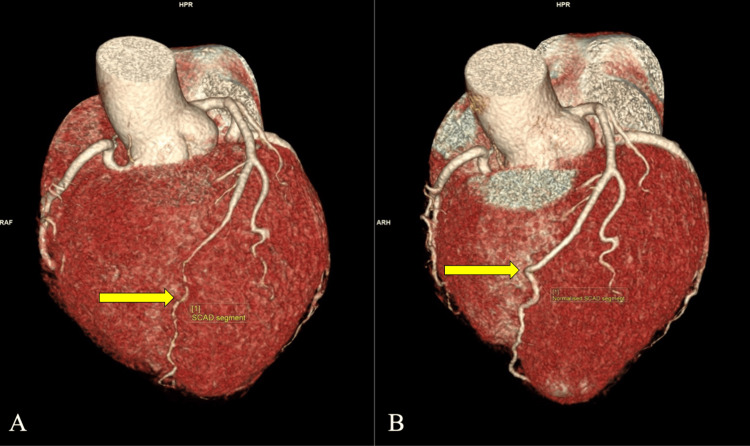
A: Reconstructed three-dimensional VRT image from coronary CT angiography showing type 2A SCAD affecting the LAD artery upon admission (arrow); B: Follow-up imaging after eight months, revealing resolution of the affected segment (arrow). VRT: Volume rendering technique; SCAD: Spontaneous coronary artery dissection; LAD: Left anterior descending.

No coronary calcium or atheromatous disease was noted. An echocardiogram was performed, which showed normal left ventricular (LV) systolic function with an ejection fraction of 60%. No regional wall motion abnormalities, significant valvular stenosis, or incompetence were noted. The patient was monitored closely and managed as per hospital protocol for ACS. She improved gradually during her stay and was deemed stable for discharge after two days. Upon discharge, she was managed conservatively with dual antiplatelet therapy (DAPT) for three months, consisting of aspirin and clopidogrel. She was additionally started on atorvastatin and bisoprolol. Plans for a follow-up and a repeat CT coronary angiography were also requested. Additionally, at the end of the three-month mark, clopidogrel was discontinued, thereby shifting to single antiplatelet therapy (SAPT) consisting of aspirin. Eight months after her initial presentation to the specialist cardiac center, she was well with no complaints. Follow-up CT coronary angiography (Figures 2C, 2D) was done and revealed no atherosclerosis or luminal narrowing. The LAD artery appears to have normal caliber with no abrupt narrowing compared to the imaging on admission. This suggests the resolution of SCAD (Figure 3).

## Discussion

SCAD is an increasingly prevailing etiology of ACS that predominantly presents in young females worldwide. It usually manifests with acute chest pain (96%) that may radiate to the left arm or neck, nausea and vomiting (24%), and SOB (20%) [2]. It is defined as a spontaneous dissection in a coronary artery, with subsequent IMH formation, causing ischemic damage with resultant infarction [2]. The pathophysiology of SCAD is characterized by the dissection between any of the three layers of the arterial wall [5]. There are two proposed mechanisms underlying the development of SCAD, most notably, the 'inside-out' and 'outside-in' hypotheses [6]. The former arises from the disruption of the intimal layer, resulting in blood flowing into the vessel wall, ultimately forming an IMH within the false lumen [5]. The 'outside-in' hypothesis describes a hemorrhage from the vasa vasorum into the medial layer of the vessel wall, forming an IMH [5]. This subsequent build-up of pressure from the IMH may also rupture into the true lumen, disrupting the intimal layer [5]. The 'outside-in' hypothesis is widely regarded as the main pathological driver of this disease [7,8]. Ultimately, both mechanisms lead to the formation of an IMH with or without intimal layer disruption that can stenose the lumen, thereby decreasing blood supply and resulting in ischemia to the supplied region [6].

The prevalence of SCAD has been difficult to quantify based on several factors, such as outdated angiographic diagnostic criteria and scarcity of clinical details [8]. It is reported to affect up to 4% of patients presenting with ACS in a single-center study and around 0.78-0.98% in larger studies [8-11]. The overwhelming majority of SCAD presentations are in women aged 44-53 years, with an incidence of 87-95% compared to males [8]. A study by Saw J et al. in North America found that 88.5% of SCAD patients were female [12]. Comparatively, a retrospective study done by Daoulah A et al. in the Middle East (ME) on SCAD prevalence in Bahrain, Saudi Arabia, the United Arab Emirates, and Kuwait found that females only comprised 51% of the total SCAD presentations [13]. This marks a drastic difference in the usual epidemiological data for SCAD, almost halving the incidence rate in females, causing them to equalize between the two sexes. This highlights the importance for physicians in the ME to keep SCAD on the differential for both sexes equally. There was also a much lower prevalence rate of SCAD in presentations of NSTEMI and STEMI in the ME, at 0.04%, compared to Western SCAD registries at 1-4%. However, the authors mention the retrospective analysis as a limitation that could have underestimated the prevalence in the ME registry [13]. In pregnant women, SCAD is the most common cause of ACS, accounting for 14-43% of MI during pregnancy [14]. Pregnancy-associated SCAD cases had a higher incidence in the ME than in Western figures at 28.5% and 2-12%, respectively [13]. A definitive cause for these differences has not yet been explained in the literature; however, differences in genetics, culture, and lifestyle are possible explanations [13]. More studies, particularly in the ME, need to be conducted to further understand and explore this discrepancy. The most common conditions that predispose to SCAD include but are not limited to FMD (31.1-45%), migraine (32.5-52%), anxiety disorder (11.7-27.8%), and hypothyroidism (11.3-26%) [2]. Triggering events are found to incite SCAD in more than 50% of patients, the most frequent being emotional stress (10-50%), isometric exercise or endurance (10-29%), Valsalva maneuver-related events (12%), and pregnancy or postpartum (4.7-16.7%) [2]. Genetic causes of SCAD have been associated with connective tissue disorders and inherited arteriopathies, manifesting in 4-10% of patients with SCAD [15]. A total of 50-75% of SCAD patients were found to concomitantly have extra-coronary arteriopathy, mainly FMD [15]. Diagnosis of SCAD is done via coronary angiography, which remains the first-line imaging modality [5]. For cases with an ambiguous diagnosis, the use of intracoronary imaging, such as intravascular ultrasound or optical coherence tomography, can aid in diagnosis [6]. Nevertheless, the use of these imaging techniques must be weighed against the risks of instrumentation in a vessel already prone to damage [6]. All coronary arteries can be implicated in SCAD; however, the LAD artery is most commonly affected, comprising 32-46% of cases [16]. In our case, CT coronary angiography was utilized to make a diagnosis that showed a dissection in the patient's LAD artery.

The Saw J et al. angiographic classification is extensively used today to differentiate the three types of SCAD [2,17]. Type 1 SCAD is classified as the appearance of contrast dye staining the arterial wall on angiography with the presence of multiple radiolucent lumens; this finding is considered pathognomonic. Type 2 SCAD is characterized by a diffuse stenotic lumen >20 mm in length. To distinguish between 2A and 2B, the former, seen in our patient, boasts the presence of unaffected vessel lumens proximal and distal to the stenotic segments. The latter is characterized by diffuse narrowing that culminates at the distal end of the artery. Type 3 closely resembles the appearance of atherosclerosis and is seen as a stenosis <20 mm [2,17]. The prevalence of each of these types varied between the Gulf and Western registries, with incidences of type 1 (51.8% and 29.1%), type 2 (42.2% and 67.5%), and type 3 (3.6% and 3.4%), respectively [13]. This reveals that the most common type in the Gulf is type 1, while type 2 prevails in Western countries. The causality between the discrepancies has not yet been established in the literature and paves the way for future research to understand this difference. Thus far, management of SCAD in stable patients remains conservative, avoiding invasive procedures such as percutaneous coronary intervention (PCI) [2,18]. This strategy is supported by medical experts and observational studies [2]. The decision to opt against conservative treatment is based on the patient's initial presenting severity, such as hemodynamic instability, total vessel occlusion, and ongoing or recurrent ischemia [6]. In comparison to Western registries, the Gulf registry was found to utilize the following approaches: medical management (40% vs. 49.7-89.7%), PCI (53% vs. 16.7-47.1%), and coronary artery bypass grafting (7% vs. 2.2-7.4%) [13]. These varying management strategies reveal a lack of global uniformity in the optimal approach. Such differences in SCAD management underscore the growing need for a more universal and standardized approach to optimize clinical outcomes worldwide. The usage of SAPT over DAPT in conservatively managed SCAD patients can play an essential role in reducing the incidence of major adverse cardiovascular events (MACE). In one study, SCAD patients were given DAPT, consisting of 100 mg of aspirin plus a P2Y12 inhibitor, or SAPT, consisting of either 100 mg of aspirin or a P2Y12 inhibitor. At follow-up, patients primarily treated with DAPT (66.3%) showed a significantly higher incidence of MACE [19]. DAPT caused an increased rate of urgent revascularization and re-infarction after SCAD presentation compared to SAPT. The findings of this study suggested that using DAPT can cause more harm than good, and opting for SAPT can reduce the overall harm associated with such therapy [18]. However, the benefit of using SAPT or DAPT in SCAD patients has not been extensively established in the literature and should be judged on a case-by-case basis [19]. Our patient was managed conservatively with DAPT for three months and later switched to SAPT. Throughout the patient's treatment course, beta-blockers were also utilized. Additionally, the use of beta-blockers appears to be protective for recurrent SCAD, reducing its recurrence by 64% over a median of 3.1 years [20,21]. However, a rigid and well-backed guideline for treating SCAD is yet to be established [20,22].

## Conclusions

SCAD remains an uncommon cause of ACS, typically presenting in younger women experiencing acute chest pain; it is also the most prevalent cause of ACS in pregnant women. Initial diagnostics are conducted through coronary angiography, which continues to be the gold standard. However, treatment is controversial, with most cases typically opting for a conservative medical approach. Our case involves a 54-year-old woman who presented with burning, non-exertional chest pain and NSTEMI on ECG. She was diagnosed with type 2A SCAD via CT coronary angiography. The management approach was conservative, resulting in favorable outcomes. This paper also highlights the significant differences in the presenting demographics and treatment of SCAD between Gulf and Western registries. A movement toward a more unified, evidence-based management plan for SCAD patients is essential for optimizing clinical outcomes globally.
